# Modelling of HIV prevention and treatment progress in five South African metropolitan districts

**DOI:** 10.1038/s41598-021-85154-0

**Published:** 2021-03-11

**Authors:** Cari van Schalkwyk, Rob E. Dorrington, Thapelo Seatlhodi, Claudia Velasquez, Ali Feizzadeh, Leigh F. Johnson

**Affiliations:** 1 The South African DSI-NRF Centre of Excellence in Epidemiological Modelling and Analysis, University of Stellenbosch, Stellenbosch, South Africa; 2 Centre for Actuarial Research, University of Cape Town, Cape Town, South Africa; 3 Centre for Infectious Disease Epidemiology and Research, University of Cape Town, Cape Town, South Africa; 4 National Department of Health, Pretoria, South Africa; 5 UNAIDS Country Office, Midrand, South Africa

**Keywords:** HIV infections, Public health

## Abstract

Globally, large proportions of HIV-positive populations live in cities. The Fast-Track cities project aims to advance progress toward elimination of HIV as a public health threat by accelerating the response in cities across the world. This study applies a well-established HIV transmission model to provide key HIV estimates for the five largest metropolitan districts in South Africa (SA): Cape Town, Ekurhuleni, eThekwini, Johannesburg and Tshwane. We calibrate the model to metro-specific data sources and estimate progress toward the 90-90-90 targets set by UNAIDS (90% of people living with HIV (PLHIV) diagnosed, 90% of those diagnosed on antiretroviral therapy (ART) and viral suppression in 90% of those on ART). We use the model to predict progress towards similarly defined 95-95-95 targets in 2030. In SA, 90.5% of PLHIV were diagnosed in 2018, with metro estimates ranging from 86% in Johannesburg to 92% in eThekwini. However, only 68.4% of HIV-diagnosed individuals nationally were on ART in 2018, with the proportion ranging from 56% in Tshwane to 73% in eThekwini. Fractions of ART users who were virally suppressed ranged from 77% in Ekurhuleni to 91% in eThekwini, compared to 86% in the whole country. All five metros are making good progress to reach diagnosis targets and all (with the exception of Ekurhuleni) are expected to reach viral suppression targets in 2020. However, the metros and South Africa face severe challenges in reaching the 90% ART treatment target.

## Introduction

The HIV/AIDS epidemic in sub-Saharan Africa is characterized by extreme geospatial variation in HIV prevalence^1–3^. Understanding this variation is critical to developing cost-effective, geographically-targeted interventions^3,4^ and to understanding the drivers of local epidemics^5,6^. In many sub-Saharan African countries, HIV prevalence is substantially higher in urban areas than in rural areas, which is thought to be a reflection of greater opportunities for sexual networking in urban centres^7^. Cities tend to attract high numbers of migrants, and migration is strongly associated with increased HIV risk^8–10^. Rates of migration are highest in young adults and men^11^, and the age and sex profile of city populations is therefore very different from that in rural areas. The high male-to-female ratios in cities are associated with higher levels of commercial sex activity, which may be important in driving high rates of HIV transmission^12–14^. Cities are often perceived to be less homophobic social environments^15^, and may therefore also have higher levels of same-sex activity. Given the rapid urbanization that is occurring in many African countries, the high levels of HIV transmission in metropolitan settings and the concentration of ‘key populations’ (sex workers, men who have sex with men and people who inject drugs) in urban centres, cities are increasingly being recognized as critical to the success of the HIV response.

In light of this, the Joint United Nations Programme on HIV/AIDS (UNAIDS) and partners launched the Paris Declaration on Fast-Track Cities Ending the AIDS Epidemic on World AIDS day in 2014^16^. On this day, the mayors of 26 cities committed to the Paris declaration and since 2014, more than 300 cities have joined. The 90-90-90 target set out in this declaration implied that 90% of people living with HIV (PLHIV) should know their status, 90% of diagnosed individuals should receive antiretroviral therapy (ART) and 90% of those who receive ART should be virally suppressed by 2020.

Some components of these targets are not simple to measure using surveillance or study data, and models have been used to estimate progress toward these targets at different scales^17^. However, at the city level, few studies have used transmission models to estimate progress^18,19^. Stuart et al.^18^ applied the Optima model^20^ to estimate progress towards and the impact of achieving the Fast-track targets in Johannesburg. The Spectrum model has been developed to provide estimates of some key HIV estimates at smaller scales^21^, but estimates at district levels are not based on dynamic transmission modelling, and rely instead on proportional disaggregation of provincial estimates (usually in proportion to population size). In addition, the Naomi model, which is currently being used to inform district-level planning in South Africa, is not a dynamic transmission model, but uses district-specific HIV prevalence and programme data to produce estimates for recent years and short-term projections^22^. The lack of dynamic transmission modelling in the Spectrum and Naomi district approaches means that they are not able to assess the impact of HIV prevention and treatment programmes on HIV incidence, or to make longer term projections.

South Africa has the largest HIV epidemic in the world, with an estimated 7.7 million people living with HIV in 2018^17^. The country has seen steady urbanization over the last three decades, with the fraction of the population residing in urban areas increasing from around 50% in 1985^23^ to 61.3% in 2018^24^. During the apartheid era, restrictions on population movement prevented black South Africans from settling permanently in ‘white’ urban centres, thus perpetuating a system of circular migration between urban and rural areas. In the post-apartheid era restrictions on population movement were lifted, but the pattern of circular urban–rural migration has persisted. The HIV epidemic in South Africa began in urban centres in the late 1980s, and early studies noted a substantially higher HIV prevalence in urban areas than in rural areas^25,26^. However, high rates of circular migration ensured a rapid transfer of HIV from urban to rural areas, and by the late 1990s and early 2000s, HIV prevalence levels were similar in urban and rural areas^27–29^. However, these crude comparisons have not controlled for age and sex differences between urban and rural areas, which could obscure the true extent of the urban–rural difference. To date, most studies of geographical variation in HIV prevalence in South Africa have focused on inter-provincial differences^6,30–33^, with few attempts to characterize differences between rural and urban, or between cities and smaller urban centres^34,35^. The same is true of studies of geographical variation in the coverage of ART and HIV prevention services^30,36,37^. The US-funded President’s Emergency Plan for AIDS Relief (PEPFAR) has prioritized HIV interventions in the 27 health districts that have the largest HIV-positive populations, which include the major metropolitan districts. Given the large fraction of the HIV-positive population in the metros, and the extent of the resulting HIV expenditure, it is important that the size of the HIV burden and performance of HIV programmes in these metropolitan districts be accurately quantified.

In this study, the Thembisa model^36^ is applied to provide HIV estimates and evaluate intervention impact in the five largest metropolitan districts of South Africa: Cape Town, Ekurhuleni (formerly known as the East Rand), eThekwini (Durban), Johannesburg and Tshwane (Pretoria). We use the model to estimate progress toward the 90-90-90 targets, as well as progress in HIV prevention. We predict progress toward 2030 targets, if interventions are maintained at current levels. Results are compared against corresponding estimates for South Africa as a whole.

## Methods

### The Thembisa model

The Thembisa model simulates the population demographics and HIV epidemic of South Africa and its provinces and has been used for a number of purposes. The model provides estimates at the national and provincial level, and the methodology of the model at both scales is described in detail in technical documents available for download at www.thembisa.org. The model stratifies the population by age, sex, risk group (the ‘high-risk’ group representing individuals with a propensity for commercial sex and/or concurrent partners), marital status, sexual experience and uptake of HIV prevention methods (condom use, male circumcision and pre-exposure prophylaxis). In addition, all adults are classified according to their HIV testing history (never tested, ever tested and ever diagnosed positive), CD4 count, initiation of ART and ART duration. The use of a wide range of different data sources in the model calibration, and the extensive validation of the model, make it the most reliable and informative model for assessing the impact of HIV in South Africa.

For this study, the Thembisa 4.2 model (the most recent version at the time this study was performed) was adapted to provide estimates at the metro level, with age- and sex-specific population size reconstructed to be consistent with estimates from the 2011 census allowing for migration projected from estimates of past migration from questions on migration in that census. With the exception of eThekwini, rates of fertility, non-HIV mortality, marriage and divorce are assumed to be the same as that of the province of the metro. In the case of eThekwini, the fertility rates were sufficiently different from the provincial rates as to require the use of metro-specific fertility rates.

The model allows for differences between metros in the uptake of traditional male circumcision and the rollout of HIV prevention and treatment services such as uptake of HIV testing, ART, prevention of mother-to-child transmission and medical male circumcision (MMC). To inform differences between metros, the model makes use of routinely reported data, as shown in the supplementary material. A Bayesian approach was adopted to fit the model to metro-specific HIV prevalence data obtained from antenatal prevalence surveys, and five general population prevalence surveys—the Human Sciences Research Council (HSRC) surveys of 2005, 2008, 2012 and 2017 and the Demographic and Health Survey (DHS) of 2016. Age-specific antenatal clinic (ANC) prevalence estimates at the metro-level are not published in the reports and therefore we did not estimate age patterns of sexual activity, but have instead used the age-specific patterns of sexual activity previously estimated at the provincial level.

We allowed for uncertainty in the following parameters: (1) the fraction of adults in the high -risk group, (2) the level of sexual mixing between high- and low-risk groups, (3) the rate of early condom use relative to national levels, (4) changes in condom use, (5) the initial HIV prevalence in high-risk women and (6) the bias in the antenatal survey data. The prior distributions for the first five parameters were the same as assumed previously at the provincial level^38^. A major source of antenatal bias is the inclusion of only public sector clinics in the survey, and therefore the prior distribution of this parameter is a function of the fraction of the population of the metro covered by medical aid (i.e. access to private healthcare). Prior distributions are summarised in Table S7.

### Estimating the first 90

In the model, PLHIV can be diagnosed in antenatal clinics, when presenting with opportunistic infections, or through other testing services. Testing depends on age, sex, stage of HIV infection and previous testing history. Metro-specific fractions of women tested during pregnancy over time were obtained from District Health Barometer reports^39^. The method of estimating the number of PLHIV who have been diagnosed is described in a previous publication^40^ and the metro-level testing data are described in the supplementary material.

### Estimating the second 90

Yearly ART initiations are estimated using Bayesian B-splines^36^. Coefficients of the splines are calibrated by comparing numbers of current ART users in the model to metro-level data. These monthly numbers were obtained from the District Health Information System (DHIS) for April 2012 to September 2018. Private sector use of ART was not available at the metro-level at the time of this analysis, and we make the assumption that the ratio of public-to-private ART use is the same as at the provincial level to inflate the public sector numbers. These derived numbers (up to September 2018) are in line with private sector data in 2019, and make up a small proportion of total ART use. As numbers of current ART users prior to April 2012 are not reliable at metropolitan district level, these numbers were approximated by applying the average ratio of metro-to-province numbers (in the period after April 2012) to the provincial numbers before April 2012.

### Estimating the third 90

The fraction of people on ART who are virally suppressed, as reported by ART programmes, may be biased due to missing data for a substantial fraction of ART patients. For the national and provincial models, a regression model was developed that estimates the ‘true’ viral suppression as a function of time, the fraction of ART patients that was tested, and the province. National and provincial data from the ART programme (DHIS) and the National Health Laboratory Service were used. For this study, we added metro-level data to the regression model. More details on the regression model are provided elsewhere^38^.

### Analysis

In addition to the targets above, we calculate the ratio of adult males to females, and the dependency ratio—defined as the ratio of dependents (children and adults aged > 65) to working age adults—in South Africa and each metro. We also calculate the reduction in new HIV infections between 2010 and 2018 to measure progress towards the target of a 75% reduction by 2020 and 90% reduction by 2030^41^.

The demographic profiles in cities are different to South Africa as a whole. In order to compare the metro-level estimates to the national-level estimates, we account for the demographic differences by standardising estimates to the age and sex distribution of the country as a whole. When estimating future levels of progress towards targets, we assume that all current interventions will be maintained, with future rates of intervention uptake equal to the average over the last 5 years for which data are available. South Africa started prescribing dolutegravir as a first-line regimen at the end of 2019, and we model the effect as an increase in viral suppression among those who use this drug^42^. In a sensitivity analysis, we investigate the impact of halving the rate of ART interruption/drop-out from 2021 onwards.

### Ethics

This mathematical modelling study used aggregated surveillance data, and approval from an institutional review board was not necessary.

## Results

**Figure 1:**
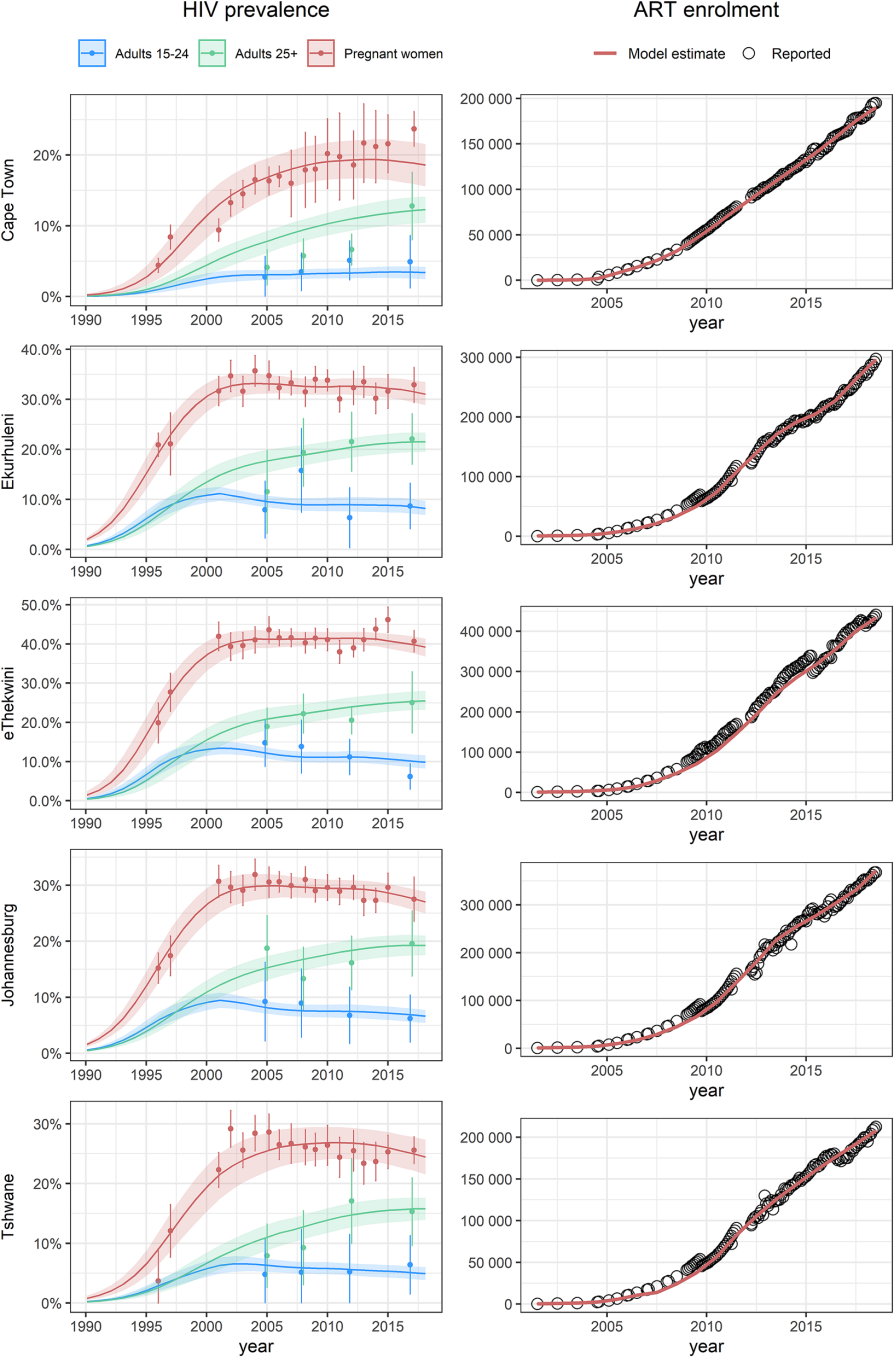
Model fits to HIV prevalence and ART enrolment data. In the left panel, dots with error bars represent survey data, and lines with shading represent the model average and 95% confidence interval. In the right panel, dots represent the reported number of clients enrolled on ART, and the red line the current enrolment in the model.

The model estimates of HIV prevalence and ART enrolment are generally consistent with the data (Fig. 1). Posterior distributions of the parameters that were varied in this calibration are shown in Table S7. In 2018, South Africa had an estimated population size of 57.3 million people, with 38.0% of this population living in the five metros in our study (Table 1). In all five metros, the adult male-to-female ratio was higher than in the country as a whole and the ratios of dependents to working age adults were lower than in the country as a whole. HIV prevalence in the reproductive age groups and overall was lower in Cape Town and Tshwane than in the country as a whole, but higher in eThekwini and Ekurhuleni, and similar in Johannesburg. When adjusting estimates to match age and sex distributions to those of the country, prevalence estimates were consistently lower than before adjustment, reflecting the greater concentration of young adults in the metropolitan districts.

**Table 1:** Demographic and HIV estimates of South Africa and its five largest metropolitan districts in 2018.

Indicator	South Africa	Cape Town		Ekurhuleni		eThekwini		Johannesburg		Tshwane	
	Estimate	Estimate	Standardised Estimate	Estimate	Standardised Estimate	Estimate	Standardised Estimate	Estimate	Standardised Estimate	Estimate	Standardised Estimate
Total population (in millions)	57.3	4.32		3.91		3.86		5.58		4.1	
Adult male to female ratio (15 +)	0.92	0.94		1.05		0.94		0.98		0.99	
Ratio of dependents (children and adults aged > 65) to working age adults	0.52	0.46		0.47		0.45		0.43		0.43	
Prevalence (total) (%)	12.9 (12.4–13.3)	8.2 (6.9–9.4)	7.5 (6.4–8.7)	14.2 (12.9–15.4)	13.1 (11.8–14.3)	16.8 (15.2–18.5)	15.9 (14.3–17.5)	12.9 (11.5–14.1)	11.3 (10–12.4)	10.2 (8.6–11.5)	9.3 (7.7–10.5)
Prevalence (15–49) (%)	18.9 (18.2–19.6)	11.8 (10–13.7)	11.2 (9.4–13)	20.4 (18.5–22.2)	19.2 (17.3–20.9)	23.8 (21.8–25.9)	23.5 (21.4–25.7)	18.1 (16.2–19.8)	16.4 (14.7–18.1)	14.3 (12.3–16.2)	13.5 (11.5–15.4)
Incidence (total) (%)	0.44 (0.4–0.48)	0.35 (0.24–0.46)	0.33 (0.23–0.44)	0.51 (0.4–0.62)	0.5 (0.39–0.61)	0.5 (0.4–0.63)	0.49 (0.39–0.62)	0.45 (0.36–0.54)	0.41 (0.32–0.49)	0.42 (0.3–0.53)	0.39 (0.28–0.5)
Incidence (15–49) (%)	0.76 (0.69–0.83)	0.59 (0.4–0.79)	0.59 (0.4–0.78)	0.84 (0.66–1.03)	0.87 (0.68–1.06)	0.84 (0.67–1.05)	0.85 (0.68–1.06)	0.71 (0.56–0.85)	0.7 (0.56–0.84)	0.67 (0.47–0.84)	0.67 (0.47–0.85)
Reduction in total incidence (2010–2018) (%)	48.7 (46.3–51.1)	36 (28.9–45.9)	34 (26.8–44.3)	49.7 (44.1–54.2)	46.2 (40.3–51.3)	55.7 (50.3–60.6)	57.2 (51.6–62.1)	50.2 (46.2–54.3)	48.2 (43.7–52.5)	38 (30.4–46.3)	37.6 (29.4–46.1)
1st 90: % of PLHIV who are diagnosed	90.5 (90–90.9)	88 (86.8–89.4)	87.1 (85.9–88.4)	89 (88–89.9)	88.8 (87.8–89.7)	92.1 (91.3–92.8)	92 (91.4–92.7)	87 (86.1–88)	86.1 (85.1–87.1)	88.2 (86.8–89.5)	87.2 (86–88.4)
2nd 90: % of diagnosed PLHIV who are on ART	68.4 (66.6–70.8)	61.4 (54.1–70.9)	61.1 (54.3–70.9)	60.2 (56.3–65.2)	60.9 (57.3–65.2)	72.5 (67.7–75.5)	72.9 (68.1–75.5)	59.5 (55.3–65)	59.6 (55.5–65)	56.4 (51.3–64.7)	56.2 (51.3–64.7)
3rd 90: % of PLHIV on ART who are virally suppressed	86.2 (84.1–88.3)	90.3 (89.0–91.6)		77.5 (72.3–82.8)		91.0 (90.0–92.1)		84.5 (82.5–86.6)		89.0 (87.8–90.2)	
ART coverage (%)	61.9 (60.2–64)	54.1 (47.2–63.2)	53.5 (47.2–61.9)	53.6 (49.7–58.5)	54.2 (50.6–58.8)	66.8 (62–69.8)	67 (62.4–69.7)	51.8 (47.8–57.1)	51.6 (47.8–56.1)	49.7 (44.8–57.7)	49.2 (44.6–56.6)

**Figure 2:**
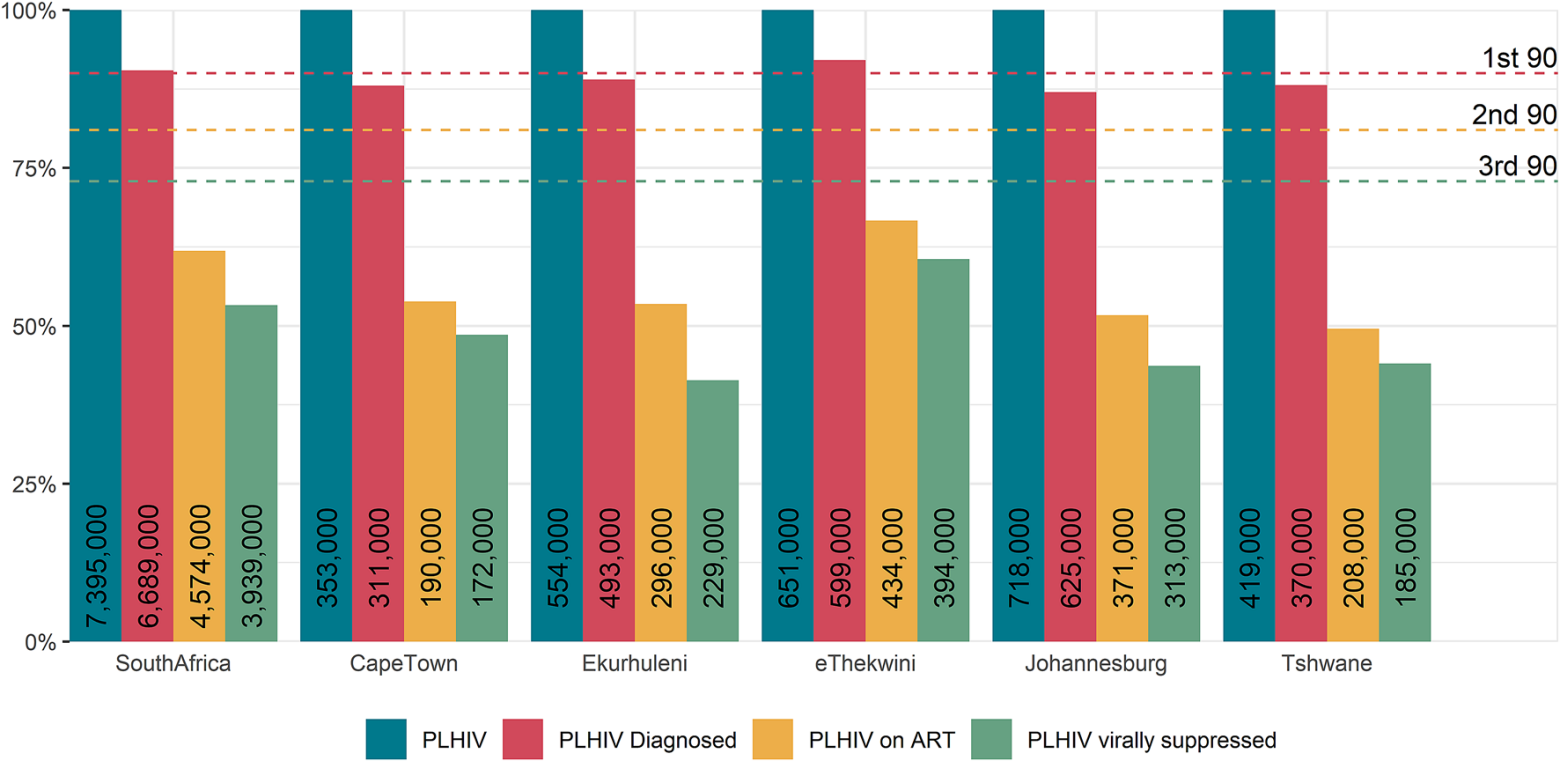
The HIV treatment cascade in South Africa and the five metropolitan districts in 2018.

Incidence follow a similar pattern to prevalence when compared to the national estimate, with highest incidence in Ekurhuleni and eThekwini. The greatest reduction in total HIV incidence between 2010 and 2018 was estimated for eThekwini (57.2%, Table 1) and the lowest reduction in Cape Town (34.0%). In 2018, 92.0% of PLHIV in eThekwini were diagnosed and therefore this metro reached the first 90% target (Table 2). The other metros lagged slightly behind the national average of 90.5%, with Tshwane having the lowest fraction of diagnosed PLHIV, at 87.2%. Progress toward reaching the second 90% target in South Africa was low, with only 68.4% of diagnosed PLHIV enrolled on ART. Cape Town, Ekurhuleni and Johannesburg lagged behind at only ~ 60% of PLHIV enrolled on ART, and Tshwane was even lower at 56.2%. In Cape Town and eThekwini, just over 90% of people enrolled on treatment were virally suppressed, with Tshwane slightly lower at 89%, Johannesburg at 84.3% and Ekurhuleni at 77.2%. eThekwini has made the best progress in terms of the fraction of its PLHIV virally suppressed, at 394,000 of 651,000 (60.5%, Fig. 2). Ekurhuleni was furthest from reaching the target, with only 229,000 of its 554,000 (41.3%) PLHIV virally suppressed.

**Table 2:** Model estimates of progress toward UNAIDS targets.

	South Africa	Cape Town	Ekurhuleni	eThekwini	Johannesburg	Tshwane
**Progress toward 90-90-90**						
By 2018	91-68-86	87-61-90	89-61-78	92-73-91	86-60-85	87-56-89
By 2020	93-73-91	89-66-93	91-68-85	94-75-94	89-72-90	89-61-93
**Progress toward 95-95-95**						
By 2030	96-78-91	94-76-94	95-76-86	97-77-94	95-77-90	95-76-93
By 2030, if ART interruption rate is halved from 2021	97-87-91	95-85-94	96-86-86	97-86-94	96-86-90	96-86-93
**Reduction in incidence from 2010**						
By 2018	48.7%	34.0%	46.2%	57.2%	48.2%	37.6%
By 2020 (target: 75%)	58.5%	42.8%	57.0%	64.6%	61.4%	48.1%
By 2030 (target: 90%)	71.9%	61.2%	71.3%	75.5%	76.4%	73.5%
By 2030, if ART interruption rate is halved from 2021	78.6%	69.3%	77.4%	82.2%	82.4%	81.1%

Confidence intervals for these point estimates are shown in Table 1 and Supplementary Table S8.

Our models estimate that none of the five metros will achieve all of the 90-90-90 targets by 2020 or the 95-95-95 targets by 2030 (Table 2). The model predicts that Ekurhuleni will be the worst performing metro, with only 76% of diagnosed PLHIV enrolled on treatment and only 86% of ART clients virally suppressed by 2030. None of the metros is likely to meet the target of reducing the number of new infections in 2010 by 75% in 2020 or 90% in 2030.

**Figure 3:**
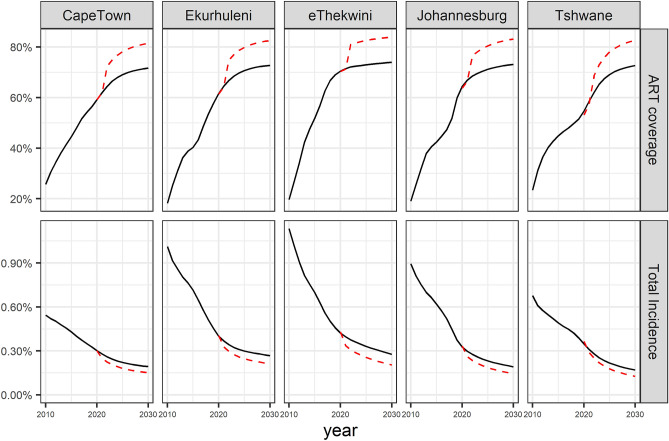
ART coverage (among adults) and total incidence if levels of HIV prevention and care are maintained (black lines) and if the estimated ART interruption rate is halved by 2021 (dashed red lines).

Reducing the rate of ART interruption by 50% by 2021 will substantially increase the fraction of diagnosed PLHIV who are on ART in 2030, from around 75% in all five metros to around 85% (Table 2). This will also substantially reduce new infections. The impact of halving the ART interruption rate on ART coverage (among adults) and total incidence is shown in Fig. 3.

**Figure 4:**
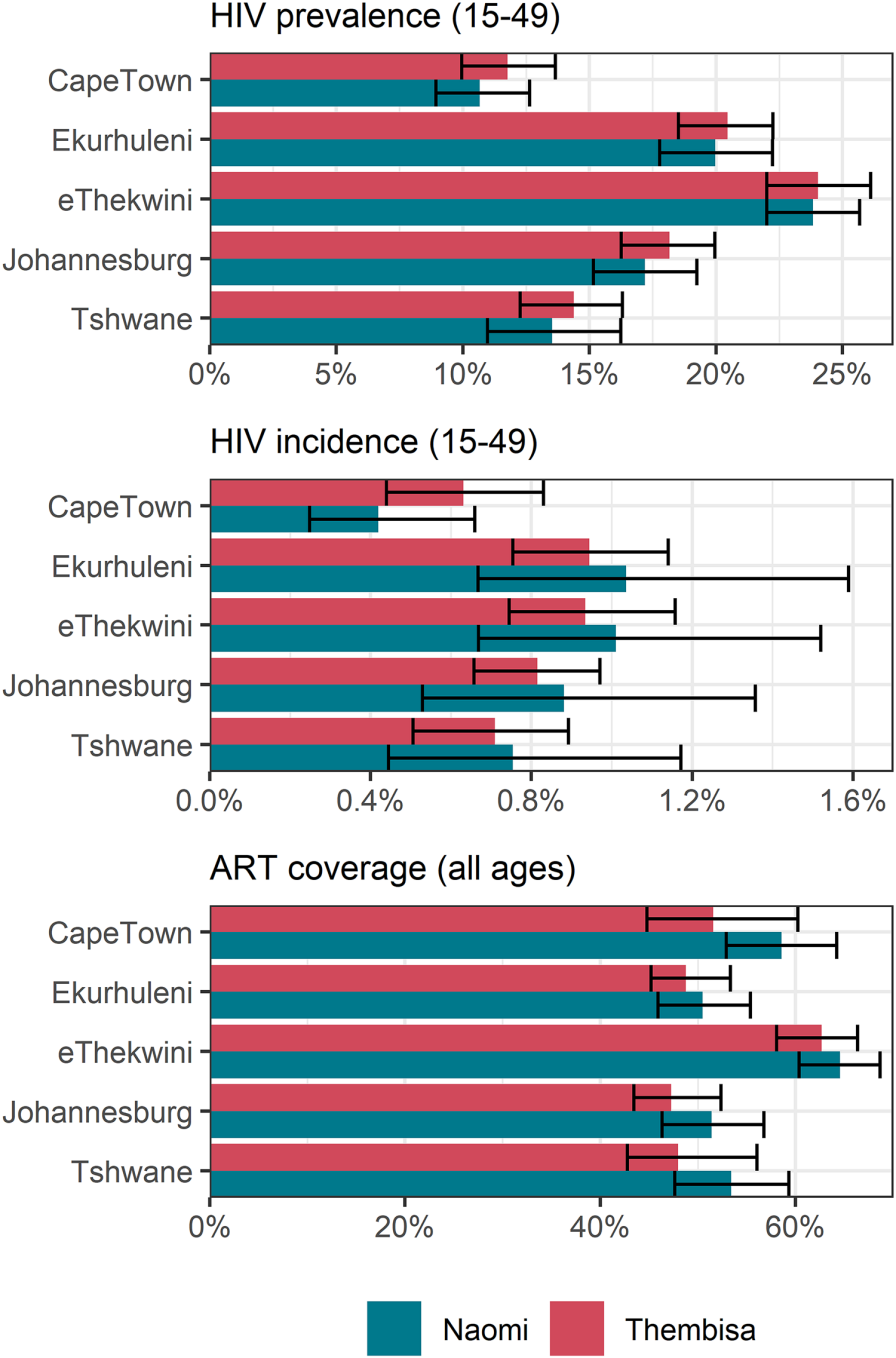
A comparison of key indicators to the Naomi model in 2017.

Thembisa estimates of HIV incidence and prevalence among 15–49 year olds, and adult ART coverage in 2017 are similar to the corresponding estimates from the Naomi model (Fig. 4). Only in Cape Town is the estimate of ART coverage substantially lower and incidence substantially higher in our model than in Naomi.

## Discussion

Around 38% of South Africa’s population and 36% of all people living with HIV live in the five metropolitan districts of Cape Town, Ekurhuleni, eThekwini, Johannesburg and Tshwane. Although there are clear differences across the metros, both in HIV prevalence and progress towards HIV targets, we do not see consistent differences when comparing these metros to the country as a whole, suggesting that urban–rural differences in HIV epidemic drivers and HIV programmes are not as substantial as previously assumed. Despite having made the best progress towards the UNAIDS targets (92-73-91) and showing the greatest reduction in incidence between 2010 and 2018 (57.2%), eThekwini still had a high incidence of 0.85% in the 15–49 year age group in 2018. Ekurhuleni is making slow progress towards enrolling on and successfully treating PLWH with ART (89-61-77), and this is reflected in the high incidence rate (0.87%) and low reduction in incidence between 2010 and 2018 (46.2%). The other two Gauteng metros are showing only slightly better progress than Ekurhuleni, at 86-60-84 in Johannesburg and 87-56-89 in Tshwane. Although Cape Town has always had a lower HIV prevalence than the other metros (Fig. 1) and had the lowest estimated incidence in 2018, the city had the lowest success in reducing incidence between 2010 and 2018, with a reduction of only 34.0%. This is likely due to relatively low uptake of MMC, lower than national levels of condom use and greater reversal of increases in condom use compared to the other metros (Table S7).

Predictions from the model show that if rates of uptake of HIV interventions are maintained at 2018 levels, none of the metros will meet all the 2020 or 2030 targets. By 2030, all the metros will have diagnosed near 95% of PLHIV, and in Cape Town, eThekwini and Tshwane nearly 95% of those on ART will have achieved viral suppression. The greatest challenge will be to enrol and retain on treatment those PLHIV who are diagnosed, with predicted levels around 75% for all five metros in 2030. If we halve the rate of ART interruption from 2021, this fraction is predicted to increase to around 85% in the five metros. Ensuring better retention of ART patients will not only improve outcomes for PLHIV, but will also have a substantial impact on reducing new infections. However, even under the optimistic assumption of a 50% reduction in the ART interruption rate, none of the five metros is predicted to reduce the number of new infections in 2010 by 90% in 2030. It is critical that interventions such as same-day ART initiation, which is associated with better ART uptake after diagnosis^43,44^, and community drug dispensing and differentiated care, which are associated with better ART retention^45,46^, are strengthened and scaled up.

This is one of the first studies to use a dynamic transmission model to estimate progress towards the targets of the Fast-Track Cities project. For South African cities, one previous published study used a model to estimate progress in Johannesburg^18^. This study estimated that 73% of PLHIV in Johannesburg were diagnosed, 66% of the diagnosed were receiving ART and 54% of those on ART achieved viral suppression in 2016. Our study’s corresponding estimates for Johannesburg in 2016 are 83-54-78. Differences may be due to the use of self-reported knowledge of status data in Optima. Our model results are very similar to those of Naomi^22^, with the only notable differences in estimates for Cape Town. Naomi incorporates household survey data on the proportion of HIV-positive individuals with detectable antiretrovirals, which are not included in Thembisa, and this may account for the generally higher estimates of ART coverage in Naomi. Since Cape Town’s ART coverage is higher in the Naomi model than in our model, HIV incidence is lower. In addition, Naomi does not consider low levels of MMC and reductions in condom use, which were more prominent in Cape Town than in the other metros in our analysis.

Ideally, routine case-based surveillance data should be used to measure progress towards targets. However, only the Western Cape province of South Africa has implemented the use of unique patient identifiers in the public health sector^47^, and it is not possible to link patient records across health services in other provinces. Even with well-functioning surveillance systems, models are still necessary to estimate the size of the undiagnosed population and the HIV incidence rate^48^. Our metro-level models have the advantage of building on models that were calibrated to national and provincial data sources, and we could rely on parameters estimated at the national/provincial level when simulating HIV dynamics in metros, where data are sparser.

However, this analysis has a number of limitations. Survey estimates that we used to calibrate our models generally lacked precision, and metro-level age-specific ANC prevalence data were not available. For this reason, metro-level age-specific sexual behaviour parameters were assumed to be the same as those of the province. We assume that the HIV profile of immigrants to a metro is the same as that of current residents (after controlling for age and sex), which may be unrealistic. We assume that the fraction of private sector ART users and private sector HIV tests are the same at metro and provincial level, which may lead to slight underestimates in the number of people on ART and number of HIV tests performed in the metros, if there is greater access to private sector HIV testing and treatment in urban settings. This model does not take into account that residents of the metro may access HIV medication and services outside the metro and vice versa, or that data for immigrants treated outside of South Africa are not included in public sector health reporting. The model does not include metro-specific data on HIV prevalence among female sex workers and men who have sex with men in calibration, although these data are available for most of the metropolitan districts. In addition, the model does not consider other key risk groups (like intravenous drug users and transgender women), which may be more common in cities and which may contribute substantially to the HIV epidemic.

Demographically, migration plays a major role in the population growth of metropolitan districts, but it is probably the parameter that is most uncertain, given the very limited data on migration since the 2011 census. The model does not take short-term fluctuations in population size (e.g. daily or weekly commuters) into account. Fertility, non-HIV mortality and marriage/divorce rates, with the exception of eThekwini-specific fertility rates, were assumed to be the same as those at the provincial level, which may bias our results. Our estimates do not reflect any uncertainty in demographic parameters. We model the impact of dolutegravir as increased viral suppression among people on ART, although evidence suggests that improved outcomes may be associated with better ART retention due to fewer side effects^49^. Regardless of the mechanism, the net effect of switching to dolutegravir is that viral suppression after ART initiation will be higher.

## Conclusion

Our results show that although the five metros are likely to reach the HIV diagnosis and viral suppression targets, the greatest challenge will be to enrol and retain diagnosed PLHIV on ART. In addition, further innovations in HIV prevention are necessary to meet the HIV incidence reduction targets.

## Supplementary Information

Supplementary Information.

## Data Availability

All data generated or analysed during this study are included in this published article (and its Supplementary Information files).
