# Supplementary material for: Modelling of HIV prevention and treatment progress in five South African metropolitan districts
Source: Sci Rep. 2021 Mar 11;11:5652. doi: 10.1038/s41598-021-85154-0 (PMC7952913; doi:10.1038/s41598-021-85154-0)
Supplement: Supplementary file 1 — Supplementary Information. [file 41598_2021_85154_MOESM1_ESM.docx]

# **Modelling of HIV prevention and treatment progress in five South African metropolitan districts**

## **Supplementary material**

Cari van Schalkwyk, Rob E. Dorrington, Thapelo Seatlhodi, Claudia Velasquez, Ali Feizzadeh, Leigh F. Johnson

This document describes data sources, assumptions and changes made to the Thembisa 4.2 provincial model [1] to produce HIV estimates for the following five South African metropolitan districts: City of Johannesburg, City of Cape Town, eThekwini, Ekurhuleni and City of Tshwane. Assumptions and parameters that are not discussed below were assumed to be the same as estimated previously at the provincial level. The provincial report (version 4.2) can be downloaded at www.thembisa.org/downloads.


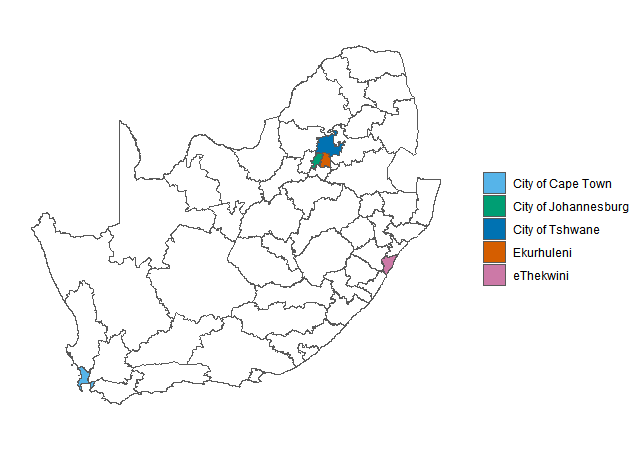


*Figure S1*: A map of South Africa showing the 5 metropolitan districts included in our study

### **Numbers of adults tested for HIV**

The National Department of Health provided total numbers of public sector HIV tests performed monthly between April 2014 and March 2018 (the financial years 2014 to 2017). In order to estimate numbers of public sector tests in the metros from 2002 to 2013, we calculate the ratio of tests performed in each metro to tests performed in the province, calculate the average of this ratio over the four years, and apply the ratio to the provincial numbers of HIV tests from 2002 to 2013.

For every year, we calculate the proportion of private sector tests at the provincial level and add this same proportion of tests to the public sector tests at metro level, due to the lack of district-specific private sector data. We assumed that the number of tests increased linearly from zero in 1990 to the estimated number in 2002.

*Table S1*: Numbers of HIV tests performed in each metro, 2002-2017

|  | **Cape Town** | **Ekurhuleni** | **eThekwini** | **Johannesburg** | **Tshwane** |
| --- | --- | --- | --- | --- | --- |
| **2002-3*** | 145 151 | 58 533 | 74 491 | 62 408 | 58 586 |
| **2003-4** | 167 484 | 68 095 | 85 949 | 72 603 | 68 157 |
| **2004-5** | 189 214 | 77 355 | 97 124 | 82 476 | 77 425 |
| **2005-6** | 137 080 | 84 386 | 134 686 | 89 973 | 84 462 |
| **2006-7** | 206 439 | 119 956 | 136 695 | 127 898 | 120 064 |
| **2007-8** | 223 537 | 149 211 | 170 459 | 159 090 | 149 346 |
| **2008-9** | 315 208 | 151 699 | 249 280 | 161 742 | 151 836 |
| **2009-10** | 572 616 | 431 296 | 572 538 | 459 851 | 431 686 |
| **2010-11** | 555 032 | 708 680 | 752 200 | 755 600 | 709 321 |
| **2011-12** | 516 729 | 659 126 | 696 160 | 702 765 | 659 722 |
| **2012-13** | 718 530 | 531 679 | 842 915 | 566 880 | 532 160 |
| **2013-14** | 623 966 | 342 422 | 686 535 | 365 093 | 342 732 |
| **2014-15** | 786 444 | 582 390 | 713 549 | 638 756 | 707 810 |
| **2015-16** | 845 897 | 737 780 | 802 221 | 790 136 | 952 669 |
| **2016-17** | 826 202 | 845 121 | 1 002 790 | 893 655 | 527 736 |
| **2017-18** | 864 729 | 846 441 | 949 938 | 877 787 | 761 185 |

*From 1 July 2002 to 30 June 2003.

### **Proportion of infants born to HIV infected mothers who are tested at 6 weeks, before 2014**

This indicator was published in the District Health Barometer [2] between 2008 and 2013. Similar to the above, we calculate the average ratio of the metro estimate to the provincial estimate over these six years and apply the ratio to the provincial estimate between 2004 and 2007.

*Table S2*: Fraction of HIV-exposed infants who are tested 6 weeks after birth

|  | **Cape Town** | **Ekurhuleni** | **eThekwini** | **Johannesburg** | **Tshwane** |
| --- | --- | --- | --- | --- | --- |
| **2004-5** | 1.9% | 1.4% | 0.8% | 2.1% | 1.5% |
| **2005-6** | 7.1% | 5.2% | 3.1% | 7.8% | 5.8% |
| **2006-7** | 15.4% | 11.3% | 6.7% | 17.1% | 12.7% |
| **2007-8** | 34.6% | 25.4% | 15.1% | 38.3% | 28.4% |
| **2008-9** | 57.2% | 31.2% | 20.5% | 66.7% | 40.9% |
| **2009-10** | 64.6% | 39.8% | 32.8% | 66.8% | 50.3% |
| **2010-11** | 74.8% | 55.9% | 53.0% | 84.7% | 56.9% |
| **2011-12** | 78.1% | 63.6% | 66.1% | 81.0% | 62.5% |
| **2012-13** | 73.8% | 84.2% | 68.5% | 98.7% | 91.6% |
| **2013-14** | 81.6% | 90.9% | 86.0% | 92.0% | 91.9% |
| **2014-15*** | 92.0% | 92.0% | 92.0% | 92.0% | 92.0% |
| **2015-16*** | 92.0% | 92.0% | 92.0% | 92.0% | 92.0% |
| **2015-16**** | 68.7% | 66% | 76.6% | 70.3% | 78.8% |

*In 2014 the methodology in calculating this fraction changed and results were implausible. Similar to the provincial and national models, we assume that 92% of HIV-exposed infants were tested in 2014 and 2015.

**In 2015, policy changed towards earlier testing – infants should now be tested at birth and again at 10 weeks. These numbers reflect the proportion of HIV-exposed infants tested at birth in 2015. After 2015, no data is available, and we assume 90% were tested at birth and 80% tested again at 10weeks – similar to the provincial models.

### **Proportion of pregnant women who receive HIV testing**

These numbers were obtained from the 2011/12 District Health Barometer [2]. Similar to the estimates above, we estimated missing values by applying the average ratio of metro to provincial estimates to the provincial estimates. We assume that 98% of pregnant women received HIV testing from 2013 onwards.

*Table S3*: Proportion of pregnant women tested for HIV

|  | **Cape Town** | **Ekurhuleni** | **eThekwini** | **Johannesburg** | **Tshwane** |
| --- | --- | --- | --- | --- | --- |
| **1999-2000** | 9.7% | 0.0% | 0.0% | 0.0% | 0.0% |
| **2000-1** | 19.5% | 3.8% | 0.0% | 6.0% | 5.0% |
| **2001-2** | 29.2% | 7.7% | 6.2% | 12.1% | 10.0% |
| **2002-3** | 46.8% | 11.5% | 17.0% | 18.1% | 15.0% |
| **2003-4** | 64.3% | 16.3% | 21.6% | 25.6% | 21.3% |
| **2004-5** | 71.2% | 24.4% | 39.1% | 38.3% | 31.8% |
| **2005-6** | 77.3% | 17.5% | 50.9% | 51.3% | 33.3% |
| **2006-7** | 83.6% | 29.8% | 57.2% | 59.0% | 43.3% |
| **2007-8** | 95.7% | 48.9% | 52.8% | 65.9% | 56.9% |
| **2008-9** | 94.7% | 64.9% | 54.7% | 70.7% | 61.3% |
| **2009-10** | 88.7% | 68.1% | 69.7% | 74.4% | 74.9% |
| **2010-11** | 86.6% | 74.2% | 77.6% | 88.9% | 82.7% |
| **2011-12** | 93.5% | 92.3% | 92.1% | 98.0% | 96.9% |
| **2012-13** | 92.5% | 79.2% | 98.0% | 87.3% | 81.9% |

### **Viral suppression**

Proportions of patients who received viral load tests and the proportions of those who were virally suppressed were obtained from the National Department of Health quarterly from the first quarter of 2015 to the first quarter of 2019. These data were added to the regression analysis as described in the provincial report and the same logic was followed to estimate viral suppression in adults and children [1]. The regression model includes national data from earlier periods in order to allow for estimation of trends in viral suppression prior to 2015. At the end of 2019, South Africa rolled out dolutegravir as a first line regimen instead of efavirenz and this drug is expected to improve rates of viral suppression [3]. Viral suppression in 2018-19 (the last year we had access to data) were adjusted for 2019-20 to account for 6 months’ use of dolutegravir, and the 2020-21 estimate reflects the full year’s use of dolutegravir. After 2020-21, viral suppression rates are assumed to be constant. Estimates for adults are shown in Table S4. These numbers were multiplied by a constant adjustment factor for paediatric viral load suppression, as described in the provincial report.

*Table S4*: Assumed proportions of adults who are virally suppressed, among adults who started ART with CD4 <200 cells/μl.

|  | **Cape Town** | **Ekurhuleni** | **eThekwini** | **Johannesburg** | **Tshwane** |
| --- | --- | --- | --- | --- | --- |
| **2005-6** | 90.0% | 73.0% | 90.9% | 82.3% | 88.4% |
| **2006-7** | 88.4% | 69.4% | 89.4% | 79.6% | 86.5% |
| **2007-8** | 86.7% | 66.2% | 87.9% | 77.1% | 84.7% |
| **2008-9** | 85.3% | 63.3% | 86.5% | 74.8% | 83.0% |
| **2009-10** | 84.0% | 61.0% | 85.3% | 73.0% | 81.6% |
| **2010-11** | 83.1% | 59.4% | 84.5% | 71.7% | 80.6% |
| **2011-12** | 82.5% | 58.5% | 83.9% | 70.8% | 79.9% |
| **2012-13** | 82.2% | 58.4% | 83.6% | 70.6% | 79.7% |
| **2013-14** | 82.5% | 59.2% | 83.8% | 71.2% | 80.0% |
| **2014-15** | 83.3% | 61.0% | 84.6% | 72.6% | 81.1% |
| **2015-16** | 84.4% | 63.5% | 85.6% | 74.6% | 82.3% |
| **2016-17** | 86.0% | 67.0% | 87.0% | 77.2% | 84.1% |
| **2017-18** | 87.7% | 71.1% | 88.5% | 80.2% | 86.1% |
| **2018-19** | 89.4% | 75.6% | 90.1% | 83.3% | 88.1% |
| **2019-20** | 91.0% | 79.9% | 91.5% | 86.2% | 90.0% |
| **2020-21*** | 92.6% | 84.2% | 93.0% | 89.0% | 91.9% |

*Assumed constant thereafter

### **Male circumcision**

Monthly numbers of medical male circumcisions (MMC) performed between 2013 and 2017 were obtained from the National Department of Health. Similar to above, we used the average ratio of metro to province for this time and applied these numbers to the provincial numbers to obtain estimated numbers of MMC performed between 2008 and 2012.

*Table S5*: Assumed numbers of medical male circumcisions occurring in each metro.

|  | **Cape Town** | **Ekurhuleni** | **eThekwini** | **Johannesburg** | **Tshwane** |
| --- | --- | --- | --- | --- | --- |
| **2008-9** | 55 | 347 | 396 | 543 | 266 |
| **2009-10** | 97 | 614 | 700 | 959 | 470 |
| **2010-11** | 155 | 4592 | 5594 | 7181 | 3521 |
| **2011-12** | 412 | 12188 | 30786 | 19058 | 9345 |
| **2012-13** | 4463 | 28260 | 34675 | 44189 | 21667 |
| **2013-14** | 5302 | 34070 | 29346 | 49578 | 28860 |
| **2014-15** | 5828 | 30524 | 38721 | 60239 | 23672 |
| **2015-16** | 5192 | 31908 | 31135 | 43099 | 21233 |
| **2016-17** | 6073 | 26159 | 28249 | 46224 | 24027 |
| **2017-18** | 9597 | 25357 | 58054 | 46068 | 23759 |

Traditional circumcision, prior to the promotion of MMC, is modelled in the same way as in the provincial model, with weighting of language groups based on the proportion of people in each language group at metro-level. Language group is used as a proxy for cultural circumcision practices. The proportions in each language group were obtained from 2011 census data [4] and circumcision practices by language group from Connolly et al. [5]. The estimated proportions of males circumcised at ages zero to forty in each of the metros are shown in Figure S2.


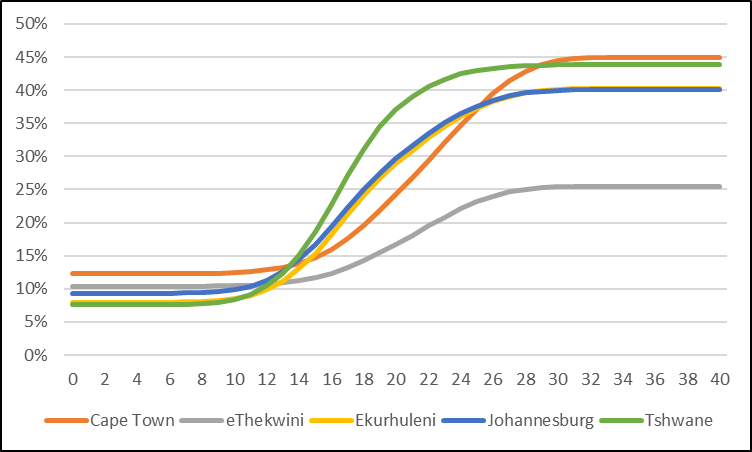


*Figure S2*: Assumed proportions of men circumcised by age, prior to the promotion of MMC

### **Prior distributions**

As described in the main text, the prior distributions for five parameters were the same as assumed previously at the provincial level [1]. In the case of the bias in the antenatal survey data, a major contributing factor is the inclusion of only public sector clinics in the survey, and therefore the prior distribution of this parameter is a function of the fraction of the district’s population that has medical aid coverage and can attend private healthcare. Estimated fractions of individuals who are members of medical aid schemes (and therefore access private healthcare) in each of the five districts were obtained from Statistics South Africa’s General Household Surveys [6] and are shown in Table S6. This information was used to derived the prior distribution of the antenatal bias parameter in the same way as at provincial level [1]. Means and standard deviations of the prior distributions, as well as the means and 95% intervals of the posterior distributions of the parameters are shown in Table S7.

*Table S6*: Fractions of the population of each district who are members of medical aid schemes

| **Year** | **Cape Town** | **eThekwini** | **Ekurhuleni** | **Johannesburg** | **Tshwane** |
| --- | --- | --- | --- | --- | --- |
| 2015 | 28.0% | 19.2% | 27.9% | 26.2% | 33.0% |
| 2016 | 28.0% | 20.6% | 26.2% | 25.9% | 33.1% |
| 2017 | 29.2% | 19.6% | 23.7% | 24.5% | 29.1% |
| 2018 | 27.7% | 20.7% | 23.1% | 21.6% | 29.6% |
| Mean: | 28.2% | 20.0% | 25.2% | 24.6% | 31.2% |
| Mean of antenatal bias parameter: | 0.577 | 0.488 | 0.544 | 0.537 | 0.609 |

### **Posterior distributions**

The posterior distributions of the sexual mixing parameter (which quantifies the level of mixing between high- and low-risk groups) appears to be similar in South Africa (0.46, 95% CI 0.43-0.49) and the districts, except in Ekurhuleni where there is less mixing between groups (0.35, 95% CI 0.23-0.52). In the national model, 35% of men and 25% of women are in the high-risk group. In Cape Town, these fractions are significantly lower (posterior mean of the adjustment factor is less than one), while the fractions in the other districts are higher than in SA. The average adjustment to condom use is below one in Cape Town, which implies that Cape Town had lower levels of condom use in the early HIV epidemic than in South Africa overall. In the other districts, the average of the condom use adjustment was slightly higher than one, but the credible intervals include one. In the model, condom use increases over time, but is allowed to decrease in more recent years since perceived risk may decrease over time. This degree of reversal would be 100% if condom use returned to 1998 levels. The posterior distributions of this risk compensation variable shows that condom use decreased more in Cape Town than in the rest of the country, but reductions were smaller in the other districts than in SA.

*Table S7*: Prior and posterior distributions of parameters varied in the model. For priors, the distribution, its mean and standard deviation is shown. For the posteriors, the mean and 95% credible intervals are shown.

| **Parameter** | **Cape Town** | | **eThekwini** | | **Gauteng metros** | **Ekurhuleni** | **Johannesburg** | **Tshwane** |
| --- | --- | --- | --- | --- | --- | --- | --- | --- |
|  | **Prior** | **Posterior** | **Prior** | **Posterior** | **Prior** | **Posterior** | **Posterior** | **Posterior** |
| Sexual mixing | Beta | 0.47 | Beta | 0.46 | Beta | 0.35 | 0.45 | 0.48 |
|  | 0.48 (0.10) | (0.30-0.67) | 0.48 (0.10) | (0.29-0.64) | 0.48 (0.10) | (0.23-0.52) | (0.30-0.62) | (0.30-0.65) |
| Antenatal bias | Gamma | 0.6 0 | Gamma | 0.59 | Gamma | 0.47 | 0.50 | 0.61 |
|  | 0.58 (0.09) | (0.47-0.75) | 0.49 (0.07) | (0.49-0.7) | 0.54-0.61 (0.03-0.08) | (0.38-0.59) | (0.40-0.62) | (0.56-0.67) |
| Multiplicative adjustment to high risk proportion in SA (35% of men and 25% of women) | Gamma | 0.55 | Gamma | 1.25 | Gamma | 1.25 | 1.09 | 1.06 |
|  | 0.61 (0.15) | (0.47-0.64) | 1.29 (0.32) | (1.09-1.46) | 1.20 (0.30) | (1.11-1.38) | (0.97-1.23) | (0.93-1.21) |
| Multiplicative adjustment to the national probability of condom use for woman aged 20 in short term relationship in 1998 | Gamma | 0.70 | Gamma | 1.02 | Gamma | 1.16 | 1.26 | 1.14 |
|  | 0.74 (0.11) | (0.49-0.91) | 1.08 (0.16) | (0.77-1.33) | 1.06 (0.16) | (0.88-1.45) | (0.97-1.57) | (0.85-1.46) |
| Degree of reversal in condom use | Uniform | 0.77 | Uniform | 0.52 | Uniform | 0.33 | 0.16 | 0.256 |
|  | 0.50 (0.29) | (0.23-0.99) | 0.50 (0.29) | (0.05-0.95) | 0.50 (0.29) | (0.02-0.89) | (0.01-0.51) | (0.02-0.66) |
| Initial prevalence among high risk women | Uniform | 0.05% | Uniform | 0.11% | Uniform | 0.27% | 0.24% | 0.04% |
|  | 0.10% (0.048) | (0.02-0.11%) | 0.20% (0.095) | (0.04-0.26%) | 0.15% (0.071) | (0.20-0.30%) | (0.15-0.30%) | (0.02-0.08%) |

*Table S8*. Demographic and HIV estimates of South Africa and its five largest districts in 2020 and 2030

| **Indicator** | **SA** | **Cape Town** | | **Ekurhuleni** | | **eThekwini** | | **Johannesburg** | | **Tshwane** | |
| --- | --- | --- | --- | --- | --- | --- | --- | --- | --- | --- | --- |
|  | **Estimate** | **Estimate** | **Standardised Estimate** | **Estimate** | **Standardised Estimate** | **Estimate** | **Standardised Estimate** | **Estimate** | **Standardised Estimate** | **Estimate** | **Standardised Estimate** |
| 2020 |  |  |  |  |  |  |  |  |  |  |  |
| Total Population (in millions) | 58.91 | 4.48 |  | 4.1 |  | 3.97 |  | 5.88 |  | 4.39 |  |
| Adult male to female ratio (15+) | 0.92 | 0.94 |  | 1.04 |  | 0.94 |  | 0.98 |  | 1 |  |
| Dependency ratio* | 0.53 | 0.47 |  | 0.49 |  | 0.44 |  | 0.44 |  | 0.44 |  |
| Prevalence (total) (%) | 12.9 (12.4-13.4) | 8.3 (7-9.5) | 7.7 (6.4-8.8) | 14 (12.7-15.2) | 13.1 (11.8-14.3) | 16.8 (15.2-18.4) | 15.9 (14.2-17.5) | 12.7 (11.5-13.9) | 11.2 (10-12.3) | 10.2 (8.7-11.4) | 9.2 (7.8-10.4) |
| Prevalence (15-49) (%) | 18.6 (17.9-19.3) | 11.7 (9.8-13.6) | 11.1 (9.3-12.9) | 20.1 (18.2-21.9) | 18.8 (17-20.6) | 23.2 (21.2-25.3) | 23 (20.8-25.1) | 17.7 (15.9-19.4) | 16 (14.3-17.6) | 14 (12.1-15.7) | 13.2 (11.3-14.9) |
| Incidence (total) (%) | 0.36 (0.33-0.39) | 0.3 (0.2-0.4) | 0.29 (0.19-0.38) | 0.4 (0.31-0.49) | 0.4 (0.31-0.5) | 0.42 (0.33-0.53) | 0.4 (0.32-0.51) | 0.33 (0.26-0.4) | 0.31 (0.24-0.37) | 0.35 (0.25-0.43) | 0.33 (0.23-0.41) |
| Incidence (15-49) (%) | 0.61 (0.55-0.67) | 0.51 (0.34-0.68) | 0.51 (0.34-0.68) | 0.67 (0.52-0.83) | 0.69 (0.53-0.86) | 0.69 (0.55-0.88) | 0.69 (0.55-0.88) | 0.52 (0.41-0.63) | 0.52 (0.41-0.63) | 0.55 (0.4-0.69) | 0.55 (0.39-0.7) |
| 1st 90: % of PLHIV who are diagnosed | 92.5 (92.1-93) | 89.9 (88.5-91.4) | 89.2 (87.9-90.5) | 91.1 (90.2-92) | 90.9 (90-91.7) | 93.8 (93.1-94.4) | 93.7 (93.1-94.2) | 89.6 (88.7-90.5) | 88.7 (87.9-89.6) | 89.8 (88.5-91.2) | 89 (87.9-90.3) |
| 2nd 90: % of diagnosed PLHIV who are on ART | 73.2 (71.9-74.7) | 65.8 (59.5-74.4) | 65.7 (59.8-74.4) | 67.6 (64-72) | 68.1 (64.7-72) | 75.1 (71.9-77) | 75.3 (72.2-77) | 71.9 (68.3-75.5) | 71.7 (68.3-75.5) | 60.8 (57.1-67.4) | 60.7 (57.2-67.4) |
| 3rd 90: % of PLHIV on ART who are virally suppressed | 90.7 (88.5-92.9) | 93.2 (91.9-94.5) |  | 85.4 (79.6-91.2) |  | 93.6 (92.6-94.7) |  | 89.9 (87.7-92.1) |  | 92.5 (91.2-93.7) |  |
| ART coverage (%) | 67.7 (66.4-69.3) | 59.1 (52.8-67.9) | 58.8 (53.1-66.6) | 61.6 (57.8-66.1) | 62 (58.5-66.1) | 70.4 (67.1-72.5) | 70.5 (67.4-72.4) | 64.4 (60.8-68.1) | 63.7 (60.3-67.2) | 54.6 (50.7-61.5) | 54.2 (50.7-60.4) |
| Reduction in Total Incidence (2010-2018) (%) | 58.5 (56.3-60.8) | 45 (38.9-54.9) | 42.8 (36.4-52.9) | 60.5 (55.8-64.5) | 57 (51.6-61.6) | 62.9 (58.1-67.4) | 64.6 (59.7-69) | 63.6 (60.3-66.7) | 61.4 (57.5-65) | 48.8 (42.4-55.1) | 48.1 (40.9-54.9) |
| 2030 |  |  |  |  |  |  |  |  |  |  |  |
| Total Population (in millions) | 66.12 | 5.17 |  | 4.9 |  | 4.48 |  | 7.08 |  | 5.58 |  |
| Adult male to female ratio (15+) | 0.93 | 0.94 |  | 1.03 |  | 0.94 |  | 0.97 |  | 1.01 |  |
| Dependency ratio* | 0.49 | 0.46 |  | 0.44 |  | 0.45 |  | 0.41 |  | 0.42 |  |
| Prevalence (total) (%) | 12.5 (11.9-12.9) | 8.4 (7-9.7) | 7.8 (6.5-9.1) | 13.2 (11.8-14.6) | 12.7 (11.3-14.1) | 15.7 (14.1-17.3) | 15.1 (13.5-16.7) | 11.8 (10.6-13) | 10.7 (9.4-11.8) | 9.8 (8.3-11) | 8.9 (7.5-10.1) |
| Prevalence (15-49) (%) | 14.1 (13.3-14.9) | 9.6 (7.7-11.4) | 9.1 (7.3-10.8) | 15.5 (13.5-17.5) | 14.8 (12.9-16.9) | 18.1 (15.9-20.4) | 17.4 (15.3-19.7) | 13.2 (11.5-14.8) | 11.9 (10.2-13.4) | 10.8 (8.9-12.6) | 10 (8.2-11.8) |
| Incidence (total) (%) | 0.24 (0.22-0.27) | 0.19 (0.15-0.23) | 0.19 (0.15-0.24) | 0.27 (0.21-0.34) | 0.27 (0.21-0.34) | 0.28 (0.22-0.34) | 0.28 (0.22-0.35) | 0.19 (0.15-0.23) | 0.19 (0.15-0.23) | 0.17 (0.13-0.21) | 0.17 (0.13-0.21) |
| Incidence (15-49) (%) | 0.38 (0.34-0.43) | 0.32 (0.24-0.39) | 0.32 (0.25-0.4) | 0.41 (0.32-0.54) | 0.43 (0.33-0.56) | 0.44 (0.35-0.54) | 0.45 (0.35-0.56) | 0.28 (0.22-0.34) | 0.29 (0.23-0.35) | 0.25 (0.19-0.31) | 0.25 (0.19-0.33) |
| 1st 90: % of PLHIV who are diagnosed | 96 (95.4-96.5) | 94.5 (93.8-95.3) | 94.2 (93.5-94.9) | 95.4 (94.7-96.1) | 95.4 (94.9-95.9) | 96.6 (96.1-97.1) | 96.6 (96.2-97.1) | 95.3 (94.7-95.9) | 94.6 (94.1-95.2) | 95.4 (94.7-96.1) | 94.9 (94.2-95.5) |
| 2nd 90: % of diagnosed PLHIV who are on ART | 77.6 (77.4-77.7) | 75.8 (75.5-76.1) | 75.7 (75.5-76.1) | 76.2 (75.9-76.5) | 76.2 (76.1-76.5) | 76.5 (76.2-76.7) | 76.6 (76.4-76.7) | 76.7 (76.5-76.9) | 76.5 (76.4-76.9) | 76.2 (75.9-76.5) | 76 (75.8-76.5) |
| 3rd 90: % of PLHIV on ART who are virally suppressed | 91.4 (89.2-93.6) | 93.6 (92.3-95.0) |  | 86.3 (80.4-92.1) |  | 94.0 (92.9-95.1) |  | 90.4 (88.2-92.6) |  | 92.9 (91.7-94.2) |  |
| ART coverage (%) | 74.4 (74-74.9) | 71.6 (70.9-72.4) | 71.4 (70.8-71.9) | 72.7 (72-73.4) | 72.8 (72.3-73.2) | 73.9 (73.4-74.4) | 74 (73.6-74.3) | 73.1 (72.6-73.7) | 72.4 (72-72.9) | 72.7 (72-73.4) | 72.1 (71.5-72.7) |
| Reduction in Total Incidence (2010-2018) (%) | 71.9 (69.8-74.1) | 64.6 (60.6-68.7) | 61.2 (56.3-66) | 73.6 (68.9-77.2) | 71.3 (65.8-75.4) | 75.6 (72.4-79.1) | 75.5 (71.9-79.1) | 78.6 (76.1-80.7) | 76.4 (73.2-78.8) | 75 (71.3-78.1) | 73.5 (69-77.1) |

*Dependency ratio= ratio of dependents (children and adults aged >65) to working age adults

## **References**

1 Johnson LF, Dorrington RE. Modelling the impact of HIV in South Africa’s provinces: 2019 update. 2019.www.thembisa.org (accessed 2 Jul 2020).

2 Massyn N, Day C, Barron P, *et al.* *District Health Barometer 2011/12*. Durban: Health Systems Trust: 2013.

3 Kanters S, Vitoria M, Doherty M, *et al.* Comparative efficacy and safety of first-line antiretroviral therapy for the treatment of HIV infection: a systematic review and network meta-analysis. *Lancet HIV* 2016;**3**:e510–20. doi:10.1016/S2352-3018(16)30091-1

4 Statistics South Africa. Census 2011. http://www.statssa.gov.za/?page_id=964 (accessed 2 Jul 2020).

5 Connolly C, Simbayi LC, Shanmugam R, *et al.* Male circumcision and its relationship to HIV infection in South Africa : Results of a national survey in 2002. *South African Med J* 2008;**98**:789–94.

6 Statistic South Africa. General Household Survey 2018. 2019.statssa.gov.za/publications/P0318/P03182018.pdf (accessed 2 Jul 2020).
